# Disparities in child mortality trends: what is the evidence from disadvantaged states in India? the case of Orissa and Madhya Pradesh

**DOI:** 10.1186/1475-9276-12-45

**Published:** 2013-06-27

**Authors:** Kim-Huong Nguyen, Eliana Jimenez-Soto, Prarthna Dayal, Andrew Hodge

**Affiliations:** 1 Centre for Applied Health Economics, School of Medicine, Griffith University, Meadowbrook, Brisbane, Queensland 4131, Australia; 2 School of Population Health, The University of Queensland, Public Health Building, Herston Road, Herston, Brisbane, QLD 4006, Australia; 3 Nossal Institute for Global Health, The University of Melbourne, Alan Gilbert Building, Barry Street, Carlton, Melbourne VIC 3010, Australia

**Keywords:** MDG 4, Under-five mortality, Neonatal mortality, Health inequalities, Orissa, Madhya Pradesh, India

## Abstract

**Introduction:**

The Millennium Development Goals prompted renewed international efforts to reduce under-five mortality and measure national progress. However, scant evidence exists about the distribution of child mortality at low sub-national levels, which in diverse and decentralized countries like India are required to inform policy-making. This study estimates changes in child mortality across a range of markers of inequalities in Orissa and Madhya Pradesh, two of India’s largest, poorest, and most disadvantaged states.

**Methods:**

Estimates of under-five and neonatal mortality rates were computed using seven datasets from three available sources – sample registration system, summary birth histories in surveys, and complete birth histories. Inequalities were gauged by comparison of mortality rates within four sub-state populations defined by the following characteristics: rural–urban location, ethnicity, wealth, and district.

**Results:**

Trend estimates suggest that progress has been made in mortality rates at the state levels. However, reduction rates have been modest, particularly for neonatal mortality. Different mortality rates are observed across all the equity markers, although there is a pattern of convergence between rural and urban areas, largely due to inadequate progress in urban settings. Inter-district disparities and differences between socioeconomic groups are also evident.

**Conclusions:**

Although child mortality rates continue to decline at the national level, our evidence shows that considerable disparities persist. While progress in reducing under-five and neonatal mortality rates in urban areas appears to be levelling off, polices targeting rural populations and scheduled caste and tribe groups appear to have achieved some success in reducing mortality differentials. The results of this study thus add weight to recent government initiatives targeting these groups. Equitable progress, particularly for neonatal mortality, requires continuing efforts to strengthen health systems and overcome barriers to identify and reach vulnerable groups.

## Background

In recent times the topic of inequalities in health services and outcomes has drawn increasing attention by scholars and policy makers alike [1,2]. While interest has historically centred on socio-economic status [3], there is increasing recognition of the importance of other dimensions, such as ethnicity and geography, in identifying disadvantage [4,5]. Yet, much is still unknown about the causes of health inequalities, a major research and policy priority [6] in developing countries seeking to attain the Millennium Development Goals (MDG) and general societal advancement.

India contributes to almost a quarter of under-five deaths and a third of neonatal deaths worldwide [7-9]. A feature of the country is disparities across its sub-populations due to social stratification, ecological-cultural diversity, and its large geographical area [10]. While inequalities in child health outcomes have been documented between the states of India and amongst broad markers within India, such as rural–urban location and wealth [11,12], less is known about equity trends within states and whether any progress in reducing inequalities has occurred of late. Amongst India’s twenty-eight states, Orissa and Madhya Pradesh (MP) rank within the top three highest states in terms of under-five mortality rates [13], with levels similar to Zimbabwe and Kenya [14]. These rates have remained persistently high over the last decade [15]. Both states have weak infrastructure compared to other states, low socio-economic status, and high levels of fertility. Subsequently, they have been included along with six other states – Bihar, Chhattisgarh, Jharkhand, Rajasthan, Uttarakhand, and Uttar Pradesh – in the Empowered Action Group (EAG) requiring high focus by the Government of India, and are also two of eighteen states targeted under the Indian Government’s flagship program for health sector reform, the National Rural Health Mission (NRHM). With a collective population of over 114 million, the two states provide fertile ground to assess the degree of health disparities across sub-populations defined by wealth and non-wealth equity markers.

In this paper, we estimated the levels and trends in under-five and neonatal mortality rates between rural–urban populations, caste/ethnicity groups, wealth categories, and sub-state districts within Orissa and MP. In India, differences in mortality risk have been linked to socioeconomic status [11,16], ethnicity [17,18], and rural/urban residential location [11,19,20], yet little is known about child mortality trends by these factors at the sub-state levels, and whether any progress in reducing disparities has occurred. While the selected equity markers are derived from the literature, our study is the first to produce levels and trends of mortality rates from 1990 at sub-state levels.

State-level inequalities are of particular importance in large, populous countries like India, where decision-making is decentralised as far down as the district level. Moreover, finer levels of disaggregation provide policy makers information on the early impacts of recent initiatives, such as the NRHM, and guidance for future programming and policies to meet the MDG 4. Additionally, this study provides crucial evidence on India’s progression towards the MDG, since persistent within-country inequalities reduce the effectiveness of the national objective of improving livelihoods.

## Methods

It has been suggested that multiple sources of data and methods are required to comprehensively capture the range and quality of information for monitoring trends and levels of inequality [21]. Using multiple data sources is believed to help mitigate reporting bias (in cases where the surveys overlap), and thus, improve the overall quality of the analysis. This argument is applicable to mortality estimation: the pooling of various results from different estimation methods can improve the overall efficiency of the results. Accordingly, in this paper, we collated all available datasets for India to estimate mortality rates.

### Ethics statement

The datasets used in this study were obtained through publicly available online resources. Full review of this study from an institutional review board was not sought as the datasets were anonymous and they are available for public use with no identifiable information on the survey participants.

### Data

The data were taken from seven datasets after a total of eight surveys were reviewed and validated. The World Health Survey was not utilised since it is only representative at the national level, and consequently, representative state-level estimation was infeasible. We utilised microdata derived from a collection of surveys, supplemented with crude death rates stemming from sample registration systems. An overview of the datasets and their usage in our study is presented in Table 1.

**Table 1:** **Overview of available datasets obtained from surveys in India**, **1990**-**2008**

**Data source**	**Year**	**Data type**		**Sample size**		**Used for equity marker**					**Comment**
				**Women**	**CEB**	**S**	**U/R**	**E**	**W**	**D**	
**DLHS**-**I**	**1998**-**1999**	SBH	Orissa	28,760	83,735	x	x	x			Converted U5MR to NMR
			MP	34,779	114,279						
**DLHS**-**II**	**2002**-**2004**	CBH	Orissa	22,134	64,879	x	x	x	x		
			MP	33,547	114,012						
**DLHS**-**III**	**2007**-**2008**	SBH	Orissa	27,842	73,639	x	x	x		x	Converted U5MR to NMR
			MP	46,148	143,532						
**DHS**-**I**	**1992**-**1993**	CBH	Orissa	3,781	12,933	x	x	x	x		Only available for Orissa, representative at state level
**DHS**-**II**	**1998**-**1999**	CBH	Orissa	3,945	12,529	x	x	x	x		Only available for Orissa, representative at state level
**DHS**-**III**	**2005**-**2006**	CBH	Orissa	3,101	9,100	x	x	x	x		Representative at state level
			MP	4,669	15,339						
**SRS**	**1971**-**2008**	Crude death rates				x	x				Data available: Orissa 1991–2008; MP 2004–2008.
**WHS**	**2003**										Not used, only representative at the national level.
**Estimation method**						Sum.	Sum.	Sum.	D	I	

*Notes*: *DLHS* District Level Health Survey, *DHS* Demographic Health Survey, *SRS* Sample Registration System, *WHS* World Health Survey, *S* State, *U*/*R* Urban/Rural, *E* Ethnicity, *W* Wealth, *D* District, *Sum* Summary Estimation, *D* Direct Estimation, *I* Indirect Estimation, *CEB* children ever born.

The first main data source was the Demographic Health Surveys (DHS) series – known as the Indian National Family Health Surveys – conducted in India in 1992–93, 1998–99, and 2005–2006. Like other DHS, these surveys provide consistent and reliable estimates of mortality and fertility, family planning, the utilization of maternal and child health care services, other related health indicators, and socio-economic measures. The sampling design was a systematic, stratified random sample of households, with two stages in rural areas and three stages in urban areas. An additional stage was undertaken in urban areas since urban wards are quite sizeable, which makes selection of households directly from a listing of all the households in a ward infeasible [22]. Unequal sample sizes were collected per state depending on the size of the state, resources available for the survey, and the desire for sub-population estimates (e.g. states, urban/rural, or metropolitan cities) [22-24]. Consequently, the national sample size, by survey standards, was unusually large. In 1992–93, a near (99%) nationally representative sample of 89,777 ever-married women aged 13–49 were interviewed from 88,562 households, with 6,254 (5,857) and 4,257 (4,602) women (households) from MP and Orissa, respectively. In 1998–99, the nationally representative sample of 89,199 ever-married women aged 15–49 from 91,196 households was collected, including 6,941 (6,749) and 4,425 (4,689) women (households) from MP and Orissa, respectively. Lastly, in 2005–06, a national total of 124,385 women aged 15–49 from 109,041 households were sampled, with 6,427 (5,488) and 4,540 (3,910) women (households) from MP and Orissa, respectively.

The second main data source used was the District Level Household and Facility Surveys (DLHS) series undertaken in 1998–99, 2002–04, and 2007–08. The DLHS is a collection of nationally representative household surveys, primarily conducted to monitor and assess the implementation and operation of the Reproductive and Child Health program across the districts of India. Similar to the DHS, the DLHS were undertaken using a systematic, multi-stage stratified sampling design and the national sample sizes are large [25-27]. For the 1998–99 wave, 474,463 currently married women age 15–44 from 529,817 households were interviewed, with 41,250 (46,355) and 28,757 (32,214) women (household) from MP and Orissa, respectively. In 2002–04, a nationally representative sample of 507,622 currently married women aged 15–44 years from 620,107 households were questioned. The number of women (households) from MP and Orissa were 38,024 (46,413) and 24,972 (31,909), respectively. Lastly, the 2007–08 survey wave covered 643,944 ever-married women aged 15–49 and 166,260 never-married women aged 15–24 from 720,320 households, with 56,574 (51,419) and 35,105 (33,172) women (households) from MP and Orissa, respectively.

The final dataset utilised by the study was the Sample Registration System (SRS) data. The SRS is a sample of birth and death registrations under the Office of the Registrar General of India, and it provides annual estimates of the population, birth rates, fertility, mortality, live births, maternal mortality, life expectancy, death rate, and other indicators at the national and state level and separately for rural and urban place of residence. Generally, the sample design adopted for the SRS is a single-stage stratified random sample [15]. The sampling frame is revised every ten years based on the latest census frame and the sample size has increased over time to approximately 1.5 million households and 7.27 million people in 2010.

In 2000, the state of Chhattisgarh was formed via the partitioning of 16 south-eastern districts of MP. As a result, the 1992–93 and 1998–99 DHS were not usable for MP since the DHS is only representative at the state level. Fortunately, given that the DLHS were representative at the district-level, we were able to map the data to fit into the structure of the newly formed states in 2000. Similarly, the SRS data were available on a yearly basis, and thus, we were able to account for the changes in the state boundaries.

The datasets were cleaned by deleting duplicates and dropping children that had unreasonable birthdays and death ages (e.g. child reported to die after the interview date). The collection of datasets resulted in a sample period from 1990 to 2007. Estimates were produced at the state level and across four equity markers: urban–rural location, ethnicity, wealth, and districts. These dimensions were selected based on the previous literature and the data that were available in order to best represent the diversity within the two states [22,27-30]. Data on the equity markers are available in all datasets, with the exception of SRS which includes measures at the state level and for rural/urban location only. As is common with health surveys, data on income and expenditure is not collected. Previous studies have utilised questions on household assets and housing characteristics to construct a wealth index using principal components analysis [31]. We follow this practice. While it is understood that there is a certain degree of correlation between these equity markers – for instance, the richest households are likely to reside in urban areas – the relative variations in mortality identified for different equity markers can reveal the main driving forces behind disparities in mortality and child health service utilisation.

### Mortality estimates

Survey measures of under-five and neonatal mortality were derived from complete birth histories (CBH) and summary birth histories (SBH) using the methods developed by Rajaratnam and colleagues [32]. Mortality from CBH were computed by pooling data from all available surveys and restructuring the datasets such that the life of each child is quantified into months of observation, where a binary variable indicates if the child is alive or died each month of the first five years of the child’s life. The pooling approach aims to mitigate some of the biases associated with CBH [32]. As outlined below, the numerous estimates were synthesised into a summary measure using Loess regression, a type of local regression. The advantages of using Loess regression are twofold [33]. First, it is a relatively simple but flexible method. Second, the method is nonparametric in the sense that it allows us to fit complex non-linear trends without *a priori* specifying a function to model the data. Hence, the data is used to determine the “correct” functional form, which is almost always unknown to a researcher.

While single-year estimates are possible, due to the relative rarity of observed deaths, precise estimates are only available over two-year periods. Using the person-month structure, survival rates, accounting for sampling weights, were computed for the age groups: 0–1 month, 1–11 months, 1–2 years, 2–3 years, 3–4 years, and 4 years-59 months. Under-five mortality rates (U5MR) are derived by combining the survival rates from all the age groups and subtracting from one, while the neonatal mortality rates (NMR) are similarly computed by subtracting the survival rate for the first age group (0–1 month) from one. In the cases where there is no other type of data available, a continuous series is created from the biennial period estimates using Loess regression, using a smoothing parameter (bandwidth) of 0.75 [34]. In such cases, confidence intervals are generated by running for each time-period/age-category 1,000 simulations of the survival probability by assuming a binomial distribution where the probability of success (*p*) is equal to the mean survival probability and the sequence *n* is equated to the number of person-months observed in the time-period/age-category. Mortality rates are then calculated for each time-period in each simulation and the 2.5th and 97.5th percentiles from the lower and upper confidence bounds for each time-period.

In the absence of CBH, under-five mortality rates were indirectly estimated from SBH using the combined method developed by Rajaratnam and co-authors that incorporates the cohort-derived and period-derived techniques into a single measure [32]. We utilised the full set of four methods available: the time since first birth cohort-derived method (TFBC), the maternal age cohort-derived method (MAC), the time since first birth period-derived method (TFBP), and the maternal age period-derived method (MAP). A combined measure was created by again applying Loess regression, and following the suggested practice, we choose an alpha value of 0.5 to smooth the data. Confidence intervals on the summary measure are computed by accounting for the parameter uncertainty in the SBH. This is achieved by performing 1,000 simulations from the multivariate normal distribution described by the point estimates and variance-covariance matrices for the coefficients in each model. From these simulated sets of coefficients we estimate under-five mortality for each data point. Loess regression is applied for each draw to combine the four methods. We calculate the standard deviation of the 1,000 Loess series and then multiply this by 4 to reflect the fact that the standard error is artificially deflated by a factor of one-quarter since we have used all the available data a total of four times. Finally, we use this corrected standard error to estimate a 95% confidence interval. This procedure was detailed by Rajaratnam and co-authors, and we introduced one modification to the process. We applied multipliers to the standard error of the summary measures to ensure that the lower bound of the uncertainty coverage was at least 95%. This more conservative approach places a larger likelihood of estimating high under-five mortality rate, resulting in wide asymmetrical confidence intervals.

To convert indirect estimates of U5MR into NMR, the relationships between U5MR and NMR rates were explored using state and sub-state direct estimates of the mortality rates from the other datasets with CBH [35]. This was achieved using three steps. First, U5MR and NMR were transformed into logit space. Second, in this space a hierarchical model with random intercept and random slope was fitted at the state and sub-state levels to relate NMR to U5MR. The model was then used to predict NMR in logit space from the indirectly estimated U5MR computed using the SBH. Finally, the NMR were transformed back into their proper values. Again this approach results in rather sizeable uncertainty bounds since a number of sources of uncertainty are taken into account (i.e. uncertainty from the CBHs, the model itself, and the SBHs estimates).

The final type of estimated U5MR was derived from the SRS. The SRS only provided under-five crude death rates aggregated across both sexes by state. Accordingly, we applied the commonly used technique outlined by Preston and co-authors to convert the crude death rates to mortality rates [36].

Having produced various estimates of under-five and neonatal mortality from the different sources and data types using either direct estimation, indirect estimation, or through conversions from crude death rates, we produced a single summary measure following the technique developed by Murray and colleagues [35], which averages all the estimators into one estimator across time, and which has been updated in recent years [7,37]. In brief, the summary measure is computed via a modified version of Loess regression that subsumes the choice of the smoothing parameter into the uncertainty, thus eliminating the need to choose a smoothing parameter. We followed the methods of Murray and colleagues with a few modifications as outlined below.

We adapted the basic model:

(1) $$1n{\;}_{5}q_{0}=\delta +\mathrm{\beta T}+\beta_{2}\mathrm{VR}+\epsilon$$

by estimating the following:

(2) $$\log\mathrm{it}\left({}_{5}q_{0}\right)=\delta +\beta_{1}\log\mathrm{it}\left(T\right)+\beta_{2}\log\mathrm{it}\left(S\right)+\epsilon$$

where _5_*q*_0_ is under-five mortality, *T* is the calendar year, *VR* is a dummy variable for vital registration system, and *S* is a dummy variable for the survey type (e.g. all DHSs are considered to be one survey type). The intercept for the final estimator is taken to be the inverse variance weighted mean of the coefficients on each dummy variable of *S*, which means that the overall level of child mortality is more heavily influenced by the more precise survey types.

We also slightly modified the standard tricubic weighting used in Loess regression procedures to ensure that useful information in the trends of child mortality is not eliminated. Specifically, let *i* denote the index of the point of interest, *j* denote the index of the point with the lowest weight, *W* denote the weight, and *D*_*j*_ denote the distance from the point of interest. Then,

(3) $$W_{i}=\left\{\begin{array}{l} W_{j}\cdot e^{-D_{j}}\\ W_{i} \end{array}\;\begin{array}{c} \mathrm{if}\;W_{i}=0\\ \mathrm{otherwise} \end{array}\right)$$

We produced simple predictions based on the last set of parameter estimates from the Loess regressions. Since the predictions for each population group within an equity marker is done independently, it allows for divergence. The predictions do not capture the possible impact achieved via intensified efforts to reduce child mortality in certain areas or by targeting specific sub-populations. Since such policies were introduced towards the end of the sample, their impacts cannot be accurately modelled.

Lastly, while the original model captured numerous sources of uncertainty – for example, the uncertainty associated with the model parameters – it does not take into account data uncertainty originating from uncertainty associated with each survey measurement, which is a measure of the accuracy of the estimator. For example, an estimator with large uncertainty will have less weight, and an estimator with small uncertainty will have more weight. In our methods, we did capture this data uncertainty. Since the final estimator’s uncertainty is already large mostly due to the smoothing parameter uncertainty, adding data uncertainty would cause the final estimator’s uncertainty to be even larger. Thus, the uncertainty coming from the choice of the smoothing parameter was eliminated – a smoothing parameter of 0.5 was chosen for equity markers with only one source of data, and a smoothing parameter of 0.25 was chosen for equity markers with multiple sources. This is not an uncommon practice as nonlinear local regression was historically developed as a tool where the user chooses the smoothing parameter and checks the stability of the results via a sensitivity analysis.

Finally, there are three issues of note. First, since in India it is known that the type of assets owned by the rural populations are different from that of the urban populations, the asset-based wealth index is derived for both rural and urban areas separately. Second, since neonatal rates are converted from indirect estimates of U5MR, in the case of the wealth groups, data restrictions imply that such rates are computed with an excessive degree of uncertainty. Consequently, we only estimated direct estimates across wealth groups, which are associated with a lower but still high degree of uncertainty. These estimates should be interpreted with some caution. Finally, only the DLHS datasets are representative at the district level. Since district boundaries have changed over time, the district estimates are produced using the most recent wave. Given the relative size of the sample, in this instance some caution is required when interpreting the results. All statistical analyses described were carried out using two statistical packages, Stata and *R*.

## Results

**Figure 1:**
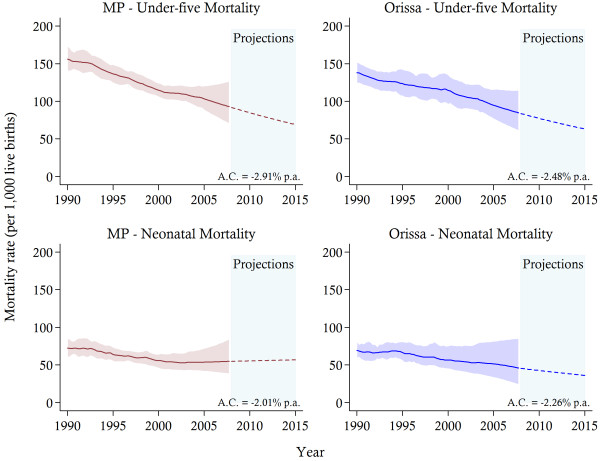
**Estimates of child mortality from 1990 and 2007 and projections towards 2015 in Madhya Pradesh (MP) and Orissa.***Notes*: The solid lines represent the mortality estimates, while the shaded area signifies 95% confidence intervals. Projections are indicated by the dotted-lines. The average annual change (A.C.) in mortality is reported.

In both states, across all the equity markers inequalities are evident. Tables 2 and 3 provide estimates of under-five and neonatal mortality rates for selected years, with corresponding confidence intervals, at the state level, and for urban–rural areas, three ethnic groupings and three wealth categories. The state-level results, reported in Figure 1, demonstrate that there has been a reduction in the U5MR in both MP and Orissa from 156 deaths per 1,000 live births (95% CI: 140–173) in 1990 to 95 (95% CI: 75–124) in 2007 and from 138 per 1,000 live births (95% CI: 124–153) to 87 (95% CI: 65–114), respectively. In comparison to recent comparable national estimates [7,37], the U5MR exceeded the national figure in both states, and the average per annum reduction over the sample period of 2.91 per cent in MP and 2.48 per cent in Orissa is slightly below the national figure of approximately 3 percent. The reductions in neonatal mortality have been even more modest. In MP, the NMR has declined from 72 per 1,000 live births (95% CI: 61–84) in 1990 to 54 (95% CI: 39–80) in 2007, while in Orissa a corresponding decline from 69 (95% CI: 59–82) to 47 (95% CI: 27–84) was observed. However, despite this overall decline, in MP, NMR has remained almost completely stagnated since 2001. If these trends continue, in Orissa by 2015 neonatal deaths will constitute more than half of all under-five deaths and over three-quarters of all infant deaths, with corresponding percentage contributions of approximately 82 and 85 in MP, respectively.

**Table 2:** **Estimated under**-**five mortality rates** (**with 95**% **confidence interval**) **for selected years**

**Equity marker**	**1990**		**1995**		**2000**		2005/2007^*^	
	**U5MR**	**95% CI**	**U5MR**	**95% CI**	**U5MR**	**95% CI**	**U5MR**	**95% CI**
**MP**	156	(141;173)	137	(124;149)	115	(106;124)	96	(75;124)
**Urban**/**Rural**								
Rural	178	(163;194)	153	(142;164)	124	(113;137)	104	(81;132)
Urban	97	(74;122)	88	(72;106)	80	(66;97)	67	(44;104)
**Ethnicity**								
Scheduled caste	186	(161;212)	163	(145;181)	134	(118;152)	107	(75;151)
Scheduled tribe	204	(174;233)	174	(158;192)	141	(124;157)	110	(77;149)
Other	130	(115;144)	118	(105;132)	99	(89;111)	85	(64;113)
**Wealth**								
*Rural*								
Low	221	(190;247)	185	(164;208)	144	(125;166)	109	(89;129)
Middle	194	(190;247)	155	(133;180)	124	(104;148)	99	(80;126)
High	130	(112;151)	107	(91;125)	87	(70;109)	70	(53;95)
**Orissa**	138	(124;153)	124	(107;143)	114	(96;135)	87	(65;115)
**Urban**/**Rural**								
Rural	150	(134;168)	127	(109;144)	120	(99;146)	92	(68;121)
Urban	98	(74;128)	87	(70;109)	79	(59;101)	68	(42;106)
**Ethnicity**								
Scheduled caste	160	(134;189)	139	(111;174)	125	(94;167)	104	(71;145)
Scheduled tribe	167	(142;196)	157	(126;196)	141	(112;176)	119	(89;156)
Other	122	(108;138)	108	(90;130)	87	(71;107)	70	(51;95)
**Wealth**								
*Rural*								
Low	166	(144;190)	144	(123;168)	121	(101;145)	100	(79;125)
Middle	151	(127;177)	131	(107;159)	113	(87;145)	95	(69;131)
High	114	(92;140)	93	(73;119)	77	(57;103)	62	(42;91)

Notes:* The estimates from the more recent year are represented in the final column. For wealth groups the most recent year is 2005, for all other equity markers the year is 2007.

**Table 3:** **Estimated neonatal mortality rates** (**with 95**% **confidence interval**) **for selected years**

**Equity marker**	**1990**		**1995**		**2000**		2005/2007^*^	
	**NMR**	**95% CI**	**NMR**	**95% CI**	**NMR**	**95% CI**	**NMR**	**95% CI**
**MP**	72	(61;84)	64	(55;74)	56	(46;64)	54	(39;80)
**Urban**/**Rural**								
Rural	81	(69;92)	69	(59;79)	59	(51;69)	58	(41;88)
Urban	53	(35;74)	50	(32;74)	45	(30;67)	42	(19;93)
**Ethnicity**								
Scheduled caste	85	(62;119)	75	(61;92)	65	(48;89)	58	(27;124)
Scheduled tribe	85	(66;105)	70	(53;90)	62	(50;78)	60	(33;109)
Other	66	(54;80)	59	(48;71)	53	(42;66)	51	(33;83)
**Wealth**								
*Rural*								
Low	89	(75;105)	75	(62;90)	63	(50;78)	52	(39;67)
Middle	89	(69;114)	75	(59;94)	62	(46;81)	51	(36;71)
High	67	(54;83)	56	(43;73)	51	(35;73)	45	(29;71)
**Orissa**	69	(59;82)	67	(56;81)	56	(45;75)	48	(27;84)
**Urban**/**Rural**								
Rural	77	(65;91)	67	(51;86)	59	(45;80)	52	(28;87)
Urban	48	(28;84)	48	(24;97)	47	(10;199)	43	(12;143)
**Ethnicity**								
Scheduled caste	83	(59;114)	72	(40;129)	66	(38;113)	56	(19;149)
Scheduled tribe	74	(53;102)	66	(43;102)	63	(35;104)	51	(23;105)
Other	64	(54;79)	64	(48;89)	51	(32;77)	48	(23;95)
**Wealth**								
*Rural*								
Low	80	(65;99)	69	(54;88)	57	(43;75)	45	(32;63)
Middle	78	(61;104)	69	(69;93)	60	(42;85)	51	(32;77)
High	67	(50;90)	55	(40;76)	45	(31;66)	37	(23;58)

Notes:* The estimates from the more recent year are represented in the final column. For wealth groups the most recent year is 2005, for all other equity markers the year is 2007.

**Figure 2:**
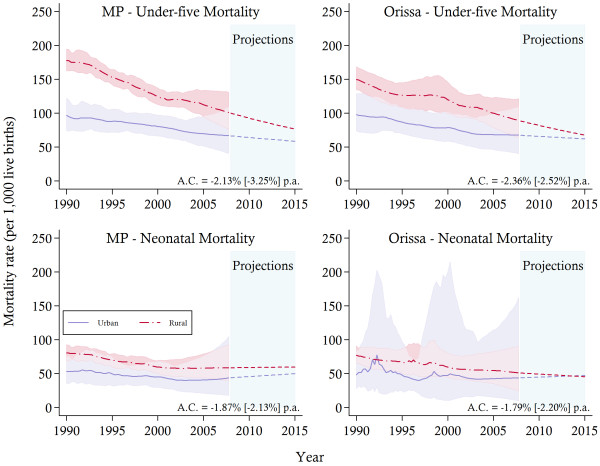
**Rural and urban child mortality trends between 1990 and 2007 and projections towards 2015.***Notes*: The solid lines represent the mortality estimates, while the shaded area signifies 95% confidence intervals. Projections are indicated by the dotted-lines. The average annual change (A.C.) in mortality is reported for urban [rural] areas. MP, Madhya Pradesh.

The general pattern of decline in under-five and neonatal mortality is observed in both urban and rural areas (see Figure 2). Children residing in urban areas remain better off than their rural counterparts in terms of the level of mortality. Yet, the rates of decline are observed to be higher in rural areas, where, for example, in MP the average annual decline in U5MR in urban areas is 2.13 per cent and 3.25 per cent in rural areas. This might point to some early outcomes of programs such as the NRHM to scale up maternal and child health coverage in rural areas. Nonetheless, at the end of the sample period the differences in mortality outcomes remained high, with the U5MR in urban areas of Orissa estimated at approximately 67 compared to 92 in rural areas. Moreover, while the gaps between rural and urban areas in both states are predicted to reduce substantially by 2015, the pattern of convergence, which is most obvious from 2003 onwards, is largely due to the inadequate progress in urban areas. In fact, the urban NMR in both states was fairly constant in the latter years of the sample, with some slight increases since 2004. However, the large uncertainty associated with the urban results, particularly in Orissa, calls for some caution when interpreting these trends.

**Figure 3:**
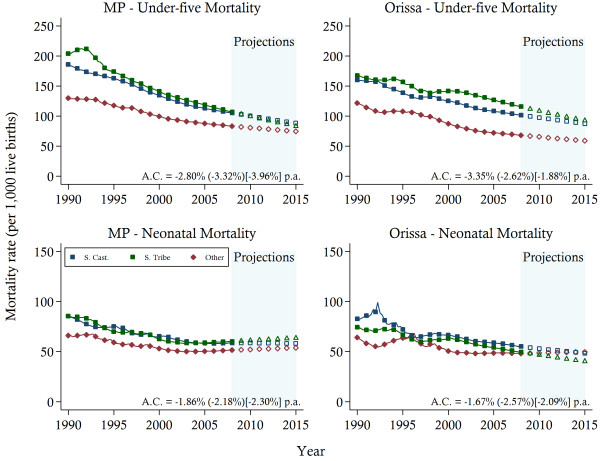
**Child mortality trends between 1990 and 2007 and projections towards 2015 by caste groups.***Notes*: The solid lines represent the mortality estimates. Projections are indicated by the hollow symbols. The average annual change (A.C.) in mortality is reported for Other (Scheduled Caste) [Scheduled Tribes] ethnic groups. S. Cast, Scheduled Caste; S. Tribe, Scheduled Tribe; MP, Madhya Pradesh.

Estimates of under-five and neonatal mortality displayed in Figure 3 confirmed higher rates among the Scheduled Castes (SC) and the Scheduled Tribes (ST) caste groups, compared to the remainder of the states’ populations (denoted as Other), although these differences are smaller for NMR as compared to U5MR. In both states, convergence between SC and ST is clearly observed. However, in MP the U5MR among the SC and the ST has fallen at a faster rate compared to that of the Other caste grouping, with average rates of reduction of 3.3 and 3.9 per cent per annum compared to approximately 2.8 per cent, respectively. In Orissa, on the other hand, the average rate of reduction has been greatest for the Other grouping, with the SC and ST experiencing an average annual decline below 2 per cent.

The differences between the ethnic groups are smaller in terms of NMR in both states. In Orissa, the estimates of NMR suggest that both the ST and the SC experienced steady reductions in mortality rates since 2000. At the same time, the NMR of the Other has stagnated at approximately 48 deaths per 1,000 live births. In MP, the performance of all ethnic groups has stagnated since 2001, again highlighting the difficulties with reducing the number of neonatal deaths in the latter years of the sample.

**Figure 4:**
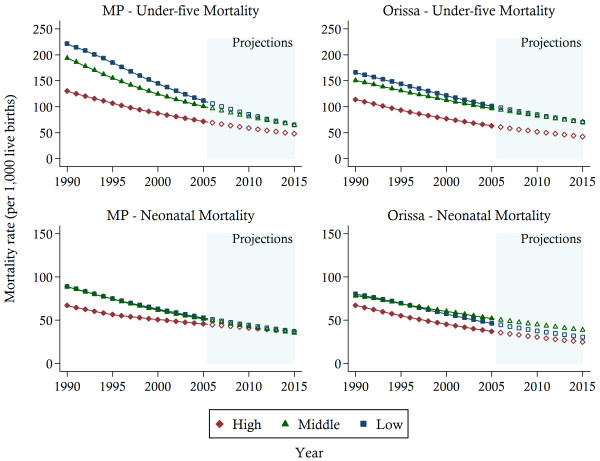
**Child mortality trends between 1990 and 2003 and projections towards 2015 in rural areas by state and three wealth groups.***Notes*: The solid lines represent the mortality estimates. Projections are indicated by the hollow symbols. High, high income; Middle, middle income; Low, low income; MP, Madhya Pradesh.

The trends in U5MR and NMR across the three wealth groups for rural areas are presented in Figure 4. While estimates were also produced for urban areas, the large uncertainty associated with the urban results (see Figure 2) and the small relative population percentage residing in urban areas (e.g. only 17% of the population reside in urban areas in Orissa), meant that further disaggregation by wealth level for the urban population yielded unstable and unreliable mortality trends, particularly for Orissa (the urban results are available upon request). Accordingly, we have opted to focus only on the wealth results derived for the rural population. The estimates suggest that all SES groups experienced mortality reduction over the past two decades. However, wealth-related disparities in mortality persist for both under-five and neonatal mortality, and are unlikely to diminish by 2015. While some convergence is observed between the Low and Middle Income groups, the High Income population continues to maintain a considerable advantage over the rest of the population. More generally, the disparities are greatest for under-five mortality in Orissa.

**Figure 5:**
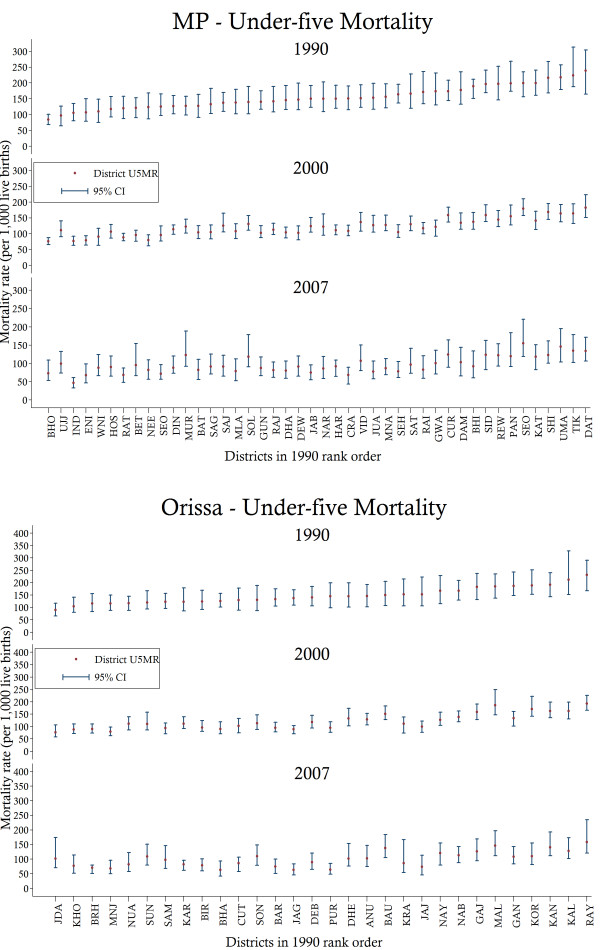
**Under**-**five mortality rates and associated confidence intervals for selected years in Madhya Pradesh (MP) and Orissa by state districts.***Notes*: District codes and names are as follows: ANU, Anugul; BAT, Balaghat; BIR, Balangir; BAR, Baleshwar; BRH, Bargarh; BAU, Baudh; BET, Betul; BHA, Bhadrak; BHI, Bhind; BHO, Bhopal; CUR, Chhatarpur; CRA, Chhindwara; CUT, Cuttack; DAM, Damoh; DAT, Datia; DEB, Debagarh; DEW, Dewas; DHA, Dhar; DHE, Dhenkanal; DIN, Dindori; ENI, East Nimar; GAJ, Gajapati; GAN, Ganjam; GUN, Guna; GWA, Gwalior; HAR, Harda; HOS, Hoshangabad; IND, Indore; JAB, Jabalpur; JAG, Jagatsinghapur; JAJ, Jajapur; JUA, Jhabua; JDA, Jharsuguda; KAL, Kalahandi; KAN, Kandhamal; KAT, Katni; KRA, Kendrapara; KAR, Kendujhar; KHO, Khordha; KOR, Koraput; MAL, Malkangiri; MLA, Mandla; MUR, Mandsaur; MNJ, Mayurbhanj; MNA, Morena; NAB, Nabarangapur; NAR, Narsimhapur; NAY, Nayagarh; NEE, Neemuch; NUA, Nuapada; PAN, Panna; PUR, Puri; RAI, Raisen; RAJ, Rajgarh; RAT, Ratlam; RAY, Rayagada; REW, Rewa; SAG, Sagar; SAM, Sambalpur; SAT, Satna; SHE, Sehore; SEO, Seoni; SOL, Shahdol; SAJ, Shajapur; SEO, Sheopur; SHI, Shivpuri; SID, Sidhi; SON, Sonapur; SUN, Sundargarh; TIK, Tikamgarh; UJJ, Ujjain; UMA, Umaria; VID, Vidisha; WNI, West Nimar. U5MR, under-five mortality; CI, confidence interval.

Finally, we reassess the spatial distribution of under-five mortality by estimating district-level estimates within both states. The results are presented in Figure 5. Two patterns are observed from these results. First, substantial variations in the levels of under-five mortality are detected in both states. In MP, the worst performing district in 1990 is found to have an U5MR of approximately 239 (95% CI: 164–304), while the best performing district achieved a rate of 84 (95% CI: 68–100). Similarly, in Orissa, the rates of under-five mortality in the worst and best performing districts are 231 (95% CI: 167–290) and 90 (95% CI: 65–117), respectively. Second, no uniform pattern of reduction is observed over time. While some of the worst performing districts in 1990 have been able to reduce their mortality rates, others continued to experience high relative levels of under-five mortality. Likewise, some of the average performing districts have been able to reduce mortality substantially while others have stagnated. Accordingly, the results suggest that geography-related inequalities persist within both states.

## Discussion

While the number of child deaths in India continues to decline at a national level [7], the disaggregation of mortality trends for two of the country’s poorest states – MP and Orissa – reveals that various sub-populations suffer disproportionately. Inequalities in under-five and neonatal mortality rates are observed between rural and urban households, social/ethnic groups, wealth strata, and sub-state governmental administrative districts. Both states have made progress in reducing high mortality rates of certain equity groups (e.g. Scheduled Tribes and rural populations). However, as a consequence, other groups have been left behind and stagnating or rising mortality levels amongst these groups has led to a lack of progress in overall U5MR and NMR. The prevalence of the within-state heterogeneity suggests that caution should be heeded when interpreting the success of nations at the aggregate level and indicates the importance of assessing the sub-national performance of countries seeking to further reduce the levels of child and maternal mortality.

Both states have shown signs of rural and urban convergence, driven primarily by the decline in rural U5MR. However further efforts are required in urban areas to see a faster rate of U5MR decline. A number of factors could be at play here. Other inequities between rural and urban populations have been documented, with those living in rural areas experiencing greater barriers to accessing health services and a higher incidence and severity of poverty [22,27]. The faster rate of decline in U5MR observed in the rural areas might be partly attributed to the success of the National Rural Health Mission and innovative policies, such as the Navjyoti scheme in Orissa, which focus on rural areas. Another possibility is that children living in urban areas, especially those who are poor, face distinct difficulties that prevent further mortality reduction, which has led to the relatively lower rate of U5MR reduction observed. For example, children and women in slums experience much higher rates of under-nutrition and anaemia than those in non-slum areas of India [38]. It has been suggested that crowding, indoor air pollution, and poor access to health services could be partially responsible for the disappointing progress in urban under-five mortality outcomes in many countries [39]. The situation is exacerbated if we also take into account the influx of the rural poor into urban slums. Figures from census data indicate almost 10 per cent of the urban population in the MP migrated from rural areas between 1991 and 2001, with livelihood opportunities a major driver of migration [40]. Increasing rural–urban migration may place upward pressure on the mortality rates of the urban segments, if poorer, low health status rural households are more likely to migrate to urban areas than better-off households. Moreover, poor immigrants lack social networks and are rarely aware of their entitlements and the availability of free or affordable health services in urban centres. The consequence is delays in seeking health care or, in some cases, no health care at all. Unfortunately, the DLHS series does not allow one to formally test for this hypothesis because they contain different waves of cross-sectional data (different households/women were surveyed for each wave; and hence, it is not possible to trace the regional movement of families). Such population pressures may have seriously affected the capacity of the health system to deliver good quality services and to achieve further improvements in mortality reduction in urban areas. Additionally, the increasing privatisation of health services in urban centres may have also posed financial barriers for the poor to good quality and affordable maternal newborn and child health services. Consequently, some caution is required when assessing the effectiveness of the rural programmes, such as the NRHM, on under-five and neonatal mortality reduction.

In Orissa, the impact of current programs aimed at reducing high under-five mortality amongst scheduled castes and tribes is obvious, but the analysis also shows that other groups are being left behind. The role of government policies could be critical in this case. For example, the ST grouping has made more progress towards the end of the sample period than the SC in reducing neonatal mortality. This might be the result of the Navajyoti scheme – launched in 2005 in 14 districts with higher-than-state-average infant mortality rates – to target tribal groups in Orissa with community-based home safe delivery and newborn care, and referrals to health centres [41]. Similarly, in MP, the converging pattern observed for U5MR may be partly attributed to state government programs aimed at improving service delivery for disadvantaged groups, such as the Deendayal Mobile Hospital Scheme, which provides outreach services to tribal areas. Other state-wide schemes, such as Bal Shakti Yojana, target malnourished children based on physical (anthropometric) measurements. The higher prevalence of under-nutrition amongst Scheduled Tribe and Scheduled Caste children could prompt greater targeting of these disadvantaged groups and could also explain some of the observed gains. Unfortunately, neither DLHS-II nor DLHS-III collects data on nutrition to enable us to examine this hypothesis.

The trends we present suggest that all socio-economic groups experienced mortality reduction over the past two decades. However, wealth-related inequality in mortality endures in rural areas. While the lowest income group has experienced rapid reduction in under-five mortality, since the mid-1990s the trends for the high income group have slowed in both states. It appears that both states have done well in targeting the poor populations in the rural sector. It should be noted, however, that many pro-poor programmes and policies were introduced in India, and in MP in particular, around 2005. Consequently, their effects on maternal and child health service utilisations and outcomes might not be fully captured by the data.

The importance of geography-related inequalities is confirmed by the district-level results. While a number of factors previously mentioned may explain the large variations between districts, we submit one additional explanation. It is reasonable to expect that the problems associated with systematic poverty and disadvantage would have stronger effects in districts where local capacity is already the weakest. The substantial demands on local governments and health departments to improve general health and living conditions are likely to weigh heavier on areas with a limited pool of resources and expertise. It is possible that the district estimates are detecting the consequences of such realities.

A noteworthy pattern observed at the state-level as well as across most of the equity markers was the stagnation of neonatal mortality rates despite higher institutional births. In fact, NMR in urban areas has increased in both states, while such rates either remained stagnant or declined very slowly in rural areas. Such findings suggest that current policies have achieved a greater impact on the level of mortality in older children (those aged 1 to 59 months), as well as on the degree of associated inequity. This is to be expected since reducing mortality in older children, unlike neonates, is more amenable to interventions that can be easily scaled up through vertical programs, such as immunisation. Interventions addressing neonatal mortality, such as basic and comprehensive emergency obstetric and neonatal care (e.g. BEmONC and CEmONC) are more complex, and therefore, depend on broad strengthening of the health systems.

In addressing the issues faced by Orissa and MP it is worthwhile identifying key factors behind the success stories of high performing states in India, many of which might lie outside the health sector. For example, in addition to a good infrastructure of health services, strong political commitment to social sectors, high levels of community awareness, and women empowerment are regarded as important drivers of the strong health indicators observed in Kerala [42,43]. Similarly for Tamil Nadu, a dynamic and accountable state government has been responsible for pioneering innovative strategies such as those related to public drug procurement, which have led to substantial improvements in the quality of health care provided to the population [44,45].

## Conclusions

In this study, we have tried to systematically collate the evidence on levels and trends in child mortality at a number of sub-national levels for two of India’s poorest states over the period 1990–2007. Nevertheless, this study has several important limitations. First, direct estimation of child mortality rates may be subject to recall bias and/or the under-reporting of deaths by mothers [35]. While we cannot deal specifically with recall bias, pooling the data from multiple surveys may mitigate recall bias to some extent where surveys overlap. Furthermore, for the periods with fewer than 10,000 person-months of observation we did not generate estimates, which limits the duration of the recall period. Second, in addition to the well-known measurement errors created by survey-based data, the limitations of indirect estimation methods have previously been documented by Rajaratnam and colleagues [32]. As they note, the main limitation of indirect methods is the need to infer information on statistics, such as the location in time of births and deaths, from observed patterns in surveys with CBH. This leads to the reliance on generalised patterns across states and across time. The impact of these generalisations is minimised by the application of local regression methods. Third, in the absence of complete vital registration systems and data on complete birth histories, we need to rely on indirect estimates of mortality rates, which led to high levels of uncertainty. In such cases, where only a limited number of observations are currently available, the large sampling errors associated with some of the trends implies caution is required when interpreting those results. Fourth, our forecasts are based on recent time trends, and consequently do not indicate the possible impact achieved via intensified efforts to reduce child mortality in certain areas or by targeting specific sub-populations. Finally, the interpretations of the various trends are speculative and require further research to exactly determine the causes for the dynamic disparities.

In conclusion, in spite of the many development challenges facing the extremely poor Indian states of MP and Orissa, both have taken great strides in reducing child mortality, both in terms of absolute levels and in the comparative disadvantage experienced by its most vulnerable groups. Nevertheless, based on the most recent data available, by 2015, MP and Orissa are unlikely to meet the national targets set by Millennium Development Goal 4. Of importance are the considerable gaps between different sub-populations, as defined by location, ethnicity, or socio-economic status. Future improvements will increasingly rely on the more difficult task of strengthening health systems and overcoming the barriers facing disadvantaged segments of the population.

## Competing interests

The authors declare that they have no competing interests.

## Author contributions

KHN, EJS and PD designed the study. AH and KHN analysed the data. EJS supervised data analyses and results reporting. PD contributed to the interpretation of results and policy implications. AH, KHN and EJS wrote the paper and all authors reviewed the manuscript. All authors read and approved the final manuscript.
